# Association of The Common CYP1A1*2C Variant (Ile462Val Polymorphism) with Chronic Myeloid Leukemia (CML) in Patients Undergoing Imatinib Therapy

**DOI:** 10.22074/cellj.2015.11

**Published:** 2015-10-07

**Authors:** Samyuktha Lakkireddy, Sangeetha Aula, Swamy AVN, Atya Kapley, Raghunadha Rao Digumarti, Kaiser Jamil

**Affiliations:** 1 Centre for Biotechnology and Bioinformatics, School of Life Sciences, Jawaharlal Nehru Institute of Advanced Studies (JNIAS), Secunderabad, Telangana, India; 2 Department of Biotechnology, Jawaharlal Nehru Technological Univesrity Anantapur (JNTUA), Ananthapuramu, Andhra Pradesh, India; 3 Department of Chemical Engineering, Jawaharlal Nehru Technological University Anantapur (JNTUA), Ananthapuramu, Andhra Pradesh, India; 4 Environmental Genomics Division, Council of Scientific and Industrial Research-National Environmental Engineering Research Institute (CSIR-NEERI), Nagpur, Maharashtra, India; 5 Department of Medical Oncology, Nizam’s Institute of Medical Sciences (NIMS), Punjagutta, Hyderabad, Telangana, India

**Keywords:** Cytochrome P-450 Enzyme System, *CYP1A1*, Polymorphism, Chronic Myeloid Leukemia, Imatinib

## Abstract

**Objective:**

Cytochrome P450 is one of the major drug metabolizing enzyme families and its role in metabolism of cancer drugs cannot be less emphasized. The association be- tween single nucleotide polymorphisms (SNPs) in CYP1A1 and pathogenesis of chronic myeloid leukemia (CML) has been investigated in several studies, but the results observed vary based on varied risk factors. The objective of this study was to investigate the risk factors associated with the CYP1A1*2C [rs1048943: A>G] polymorphism in CML patients and its role in therapeutic response to imatinib mesylate (IM) affecting clinico-pathological parameters, in the Indian population.

**Materials and Methods:**

In this case-control study, CYP1A1*2C was analysed in CML patients. After obtaining approval from the Ethics Committee of oncology hospital, we collected blood samples from 132 CML patients and 140 matched controls. Genom- ic DNA was extracted and all the samples were analysed for the presence of the CYP1A1*2C polymorphism using allele-specific polymerase chain reaction, and we examined the relationship of genotypes with risk factors such as gender, age, phase of the disease and other clinical parameters.

**Results:**

We observed a significant difference in the frequency distribution of CYP1A1*2C genotypes AA (38 vs. 16%, P=0.0001), AG (57 vs. 78%, P=0.0002) and GG (5 vs. 6%, P=0.6635) between patients and controls. In terms of response to IM therapy, significant variation was observed in the frequencies of AA vs AG in major (33 vs 67%) and poor (62 vs 31%) hematological responders, and AA vs AG in major (34 vs. 65%) and poor (78 vs. 22%) cytogenetic responders. However, the patients with the GG homozygous genotype did not show any significant therapeutic outcome.

**Conclusion:**

The higher frequency of AG in controls indicates that AG may play a protec- tive role against developing CML. We also found that patients with the AG genotype showed favorable treatment response towards imatinib therapy, indicating that this polymorphism could serve as a good therapeutic marker in predicting response to such therapy.

## Introduction

Chronic myeloid leukemia (CML) is an acquired hematopoietic stem cell disease, characterized by an increased production of immature granulocytes (blasts) which accumulate in the bone marrow and interfere with the normal blood cell production, accounting for 30% of adult leukemias (1). The symptoms of CML include bone marrow hypercellularity, anaemia, splenomegaly and leucocytosis (1). CML progresses slowly in three phases of chronic phase (CP), accelerated phase (AP) and blast phase (BP) which are differentiated by the number of blast cells in the blood and the bone marrow, and the severity of the symptoms. In 95% of CML cases, chromosomal translocation resulting in the formation of the Philadelphia (Ph) chromosome is observed (2, 3), which in turn leads to the formation of the **BCR-ABL** fusion gene. This reciprocal translocation, creating an elongated chromosome 9 der(9) and a truncated chromosome 22 (Ph chromosome), is the hallmark of CML and the main drugs which target this oncogenic fusion gene (**BCR-ABL**) are imatinib and sunitinib. The protein encoded by the fusion gene exhibits enhanced tyrosine kinase activity and plays a key role in the initiation and maintenance of CML by activating various intracellular signalling pathways resulting in uncontrolled proliferation, decreased apoptosis and survival of leukemic stem cells (4).

Since the *ABL* gene expresses a membraneassociated tyrosine kinase protein, the **BCR-ABL** transcript is also translated into a tyrosine kinase. Although the activity of this enzyme is typically controlled by other molecules, the mutant tyrosine kinase encoded by the **BCR-ABL** transcript results in a protein that is "always on" or constitutionally expressed, resulting in unregulated cell division (i.e. cancer). Though the BCR region also codes for serine/threonine kinases, the tyrosine kinase activity is more pertinent for drug therapy. The tyrosine kinase inhibitor imatinib mesylate (IM, Gleevec^®^), the CML drug of choice, inhibits the **BCR-ABL** tyrosine kinase function in Ph (Ph+) cells, causing functional alterations in genes involved in cell cycle control and cell adhesion, and ultimately results in the death of Ph+ cells via apoptosis . It is most effective in treating patients in the CP phase and controls the disease in about 75% of these patients (5, 6). However, in some cases, resistance develops after an initial favourable response and in some CML-CP patients it is not effective at all (7). Though the exact mechanism underlying the drug resistance is not yet clear, it has been suggested that additional acquired chromosomal aberrations and mutations in **BCR-ABL** kinase domain may be responsible for this resistance (8, 9). Patients in the advanced phases of AP and BP may not respond to the drug due to the acquisition of various molecular abnormalities in undifferentiated leukemic cells (10).

The genetic variability single nucleotide polymorphisms (SNPs) in genes encoding phase I and phase II drug metabolizing enzymes which detoxify the xenobiotics [xenobiotic metabolizing enzymes (XMEs)], have been linked with the variation in susceptibility of different individuals toward leukemia (11, 12) as well as with therapeutic response of individuals toward drugs (13). Our previous work on SNPs in genes encoding drug metabolizing enzymes and drug transporters showed that these polymorphisms affect drug response in breast cancer (14-16), and head and neck cancer (17).

Cytochrome P450, family 1, Subfamily A, polypeptide 1 (*CYP1A1*), a polymorphic gene which codes for the important phase-I XME aryl hydrocarbon hydroxylase, is involved in drug metabolism and activation of a number of exogenous procarcinogens (18) into highly reactive electrophilic carcinogenic molecules. These electrophiles can bind to DNA and form adducts, leading to mutations in tumor suppressor genes and proto-oncogenes, thus initiating carcinogenesis if not repaired by the DNA repair system. Hence, this gene may play an important role in both the etiology of cancers and as a determinant of cancer therapy response (19, 20).

Three single nucleotide polymorphisms of *CYP1A1* have been studied in relation to various cancers, namely T6235C (*2A), A4889G (*2C) and C4887A (*4). CYP1A1*2C SNP A4889G (rs1048943; exon 7) (21, 22) leads to the substitution of Isoleucine (Ile) by Valine (Val) at position 462 in the protein, which results in a two-fold higher catalytic activity and mutagenicity due to its greater hydrophobicity. The association of the CYP1A1*2C polymorphism with increased susceptibility to acute lymphoid leukemia (ALL) has been reported (23). Contradictory reports are present in the literature about the association of CYP1A1*2C in ALL (24) and AML (25) patients, however, not much is known about its relation with CML except a Turkish cohort study reporting that SNPs in this gene are associated with the risk of CML (26). More importantly, to our knowledge, no reports are available on the association of this polymorphism with the CML risk in the Indian population. So, we hypothesized that this SNP in *CYP1A1* may act synergistically with the **BCR-ABL** fusion oncogene in causing CML. This means that the individuals carrying SNPs in the *CYP1A1* gene are at higher risk of developing CML than those who do not carry the SNP. The CYP1A1*2C polymorphism has also been reported to vary in frequency depending on the ethnicity. For instance, this polymorphism was less prevalent in Iran and was thus suggested to play no role in CML development in Iranian patients (27), however, it was found to be involved in the Turkish population (26). *CYP1A1* may also play a role in imatinib metabolism (28). In view of such conflicting results, we have conducted a case-control study in the Indian population to examine the association of CYP1A1*2C polymorphism with CML risk and whether this polymorphism affects the therapeutic response of the patients to Imatinib.

## Materials and Methods

In this case-control study, the association of CYP1A1*2C with CML was analyzed. Approval for the study was obtained from the Ethics Committee of the Hospital (Bhagwan Mahavir Medical Research CentreHyderabad, Oncology hospital) and informed consent was obtained from all participants by explaining the importance of the study.

### Study population and their stratification

132 patients diagnosed with CML and 140 healthy age and sex matched controls formed our study group. The cohort of patients consisted of 76 (58%) male and 56 (42%) female patients with a mean age of 37.5 years. In addition, 102 (77%) patients were in the chronic phase, 20 (15%) patients were in the accelerated phase and 10 (8%) patients were in the blast crisis phase. CML patients were diagnosed at the Department of Medical Oncology, Nizam’s Institute of Medical Sciences (NIMS), Hyderabad, India. The control group consisted of individuals without the history of cancer and other diseases like genetic disorders, allergy, asthma, etc.

### Inclusion and exclusion criteria

In this study, fully diagnosed CML patients receiving Imatinib treatment (n=129) and few CML patients prior to Imatinib treatment (n=3) were included as cases. Age range of the CML patients was 14-60 years in females and 15-64 years in males. The diagnosis of CML was based on the standard clinico-hematological criteria by detecting the Ph chromosome (karyotyping) and/or the **BCR-ABL** fusion gene by reverse transcriptionpolymerase chain reaction (RT-PCR). The exclusion criteria included the patients suffering from any other disease such as chronic myelomonocytic leukemia and other myeloproliferative disorders.

### Clinico-pathologic data

Patient’s data including occupational history, complete clinical examination and routine laboratory tests such as complete blood picture [white blood cell (WBC) count and platelet count], liver and kidney functions were obtained. In addition, clinical data including phase of the disease and response to imatinib therapy were collected from the medical records with the permission of the attending medical oncologist. Patients were stratified based on response according to the European LeukemiaNet (ELN) criteria. Response status of patients to imatinib therapy (hematological, cytogenetic) was classified on the basis of WBC count, percentage of Ph+ cells and the duration of response to imatinib therapy (29, 30). We stratified the patients in our study based on these parameters to examine the efficacy of their treatment regimen. The effect of imatinib treatment was assessed after 3, 6 and 12 months. An ideal treatment regimen leads to a major/complete hematological response (MHR) and at least a minor cytogenetic response (mCyR) within 3 months, a partial CyR (PCyR) within 6 months and a complete CyR (CCyR) within 12 months

### New method of stratification of chronic myeloid leukemia patients

We established one more criterion of stratification of the CML patients, with reference to imatinib therapy, based on the genotypes of the drug metabolizing enzyme gene *CYP1A1* and its role in response to the treatment. For this reason, we carried out genomic studies for the gene *CYP1A1* using samples from both the patient and control groups.

### DNA isolation

Venous blood samples (5 ml) from patients diagnosed with CML and treated with imatinib therapy, and from control individuals were collected in ethylenediamine tetraacetic acid (EDTA, BD, India) coated vacutainers. Genomic DNA was isolated by a standard salting out method (31) and then stored at -20˚C until use for mutational analysis.

### Genotyping and the detection of CYP1A1*2C

To detect the CYP1A1*2C polymorphism, an allelespecific PCR was performed using two sets of primers. Primers were purchased from Bioserve (Hyderabad, India), dNTPs and Taq polymerase from Labpro (India). Two allele-specific forward primers (F1P- 1A1A; 5ˊ-GAAGTGTATCGGTGAGACCA-3ˊ and F2P-1A1G; 5ˊ-GAAGTGTATCGGTGAGACCG- 3ˊ), were used for PCR amplification together with a reverse primer (RP-1A1.1 5ˊ-GTAGACAGAGTCTAGGCCTCA-3ˊ). Two amplification reactions were needed for each individual analysed, one with primers 1A1.1 (common reverse primer)/1A1A (forward primer-1, specific for Ile allele) and the other with primers 1A1.1 (common reverse primer)/1A1G (forward primer-2 specific, for Val allele) which recognize the Val462 allele. The PCR reactions were performed in a final volume of 25 μl containing 50-150 ng of genomic DNA, 5 μl of 10X PCR buffer containing 20 mM MgCl_2_ (Bioserve, India), 0.5 μl of 10 pmol of each primer, 0.5 μl of dNTPs mix (200 μM), 1 U/μl of taq DNA polymerase and deionized water. The PCR cycling conditions were an initial denaturation at 94˚C for 3 minutes, followed by 30 cycles of denaturation at 94˚C for 30 seconds, annealing at 65˚C for 45 seconds and extension at 72˚C for 1 minute along with a final extension at 72˚C for 10 minutes in a Veriti^TM^ 96 well thermal cycler (Applied Biosystems, USA).

### Statistical analysis

The relationship between the CYP1A1*2C polymorphism and the development of CML with respect to the clinical characteristics was examined by using odds ratio (OR) and 95% confidence intervals (CI) derived from the logistic regression analysis using MedCalc version 7.4.1.0 (MedCalc software, Mariakerke, Belgium). P values of less than 0.05 were regarded as statistically significant.

## Results

The mean age of CML patients was 36.7 years in females and 38.3 years in males. The CML patients were divided into 4 groups according to their age at diagnosis (i.e., <20, 20-30, 31-40 and 41-60 years). Incidence of CML was found to be highest in the age group 21-30 (39%), followed by 41-60 years (34%). In contrast, the incidence was found to be low in the age group 31-40 years (22%) and lowest in those less than 20 years (5%). This indicates that the onset of CML is generally after 20 years of age.

### CYP1A1*2C genotyping analysis

**Fig.1:**
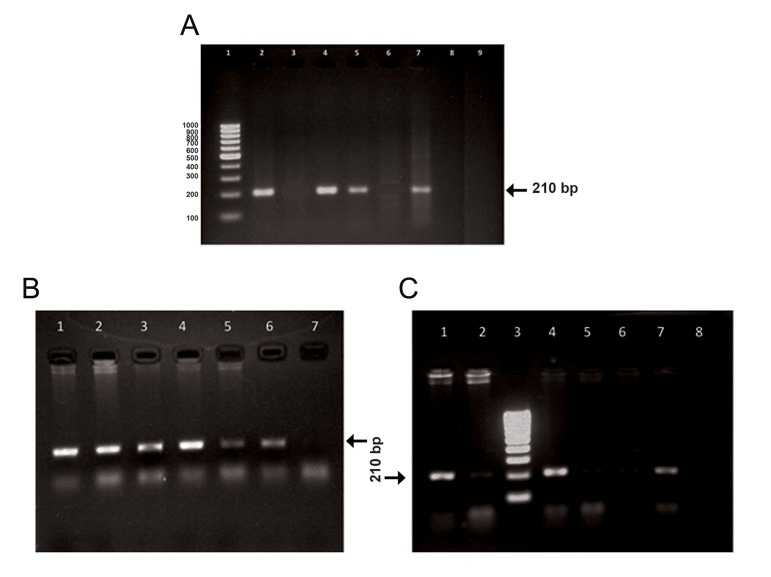
Gel electrophoresis of CYP1A1*2C PCR products using allele-specific primers. A. Lane 1 represents the 100 bp DNA marker. Lanes 2, 3 correspond to the Ile/Ile homozygous wild type individuals, lanes 4, 5 correspond to the Ile/Val heterozygous individuals and lanes 6, 7 show Val/Val homozygous mutant genotype. Lanes 8, 9 correspond to the negative controls, B. Lanes 1, 2 correspond to the Ile/Val heterozygous mutant genotype, lane 3 represents the positive control, lanes 4, 5 correspond to the Ile/Val heterozygous mutant genotype, lanes 6, 7 correspond to Ile/Ile homozygous wild type genotype and C. Lanes 1, 2 correspond to the Ile/Val heterozygous mutant genotype, lane 3 represents the 100 bp DNA marker, lanes 4, 5 correspond to the Ile/Val heterozygous mutant genotype, lanes 6, 7 correspond to the Ile/Val heterozygous mutant genotype and lane 8 represents the negative control. PCR; Polymerase chain reaction.

CYP1A1*2C gene polymorphism was genotyped by identifying both the wild type Ile allele (210 bp DNA fragment with the 1A1A1/1A1.1 primers) and the mutant Val allele (210 DNA fragment with 1A1G/1A1.1 primers) (Fig .1).

The distribution of CYP1A1*2C polymorphism genotypes in CML patients and in control individuals is shown in table 1. The frequencies of *CYP1A1* Ile/Ile (A4889A), Ile/Val (A4889G), and Val/Val (G4889G) genotypes were 38, 57, and 5% in CML patients and 16, 78, and 6% in controls respectively. We observed differences in the distribution of *CYP1A1* Ile/Ile wild type and Ile/Val heterozygous genotypes between patients and controls in the study population. Our analysis of the frequency distribution of genotypes between patients and controls showed that the AG genotype was lower in CML patients (95% CI=0.2176 to 0.6296, OR=0.3701, P=0.0002) when compared with the controls, suggesting that this genotype may play a protective role in reducing CML risk in our cohort. Further, the distribution of homozygous wild type Ile/Ile genotype was found to be higher in the CML patients (38%) than that in the controls (16%) (95% CI=1.84 to 5.8133, OR=3.2705, P=0.0001). We did not find any significant difference in the distribution of GG genotype between patients (5%) and controls (6%) (95% CI=0.265 to 2.33, OR=0.7857, P=0.6635) (Table 1). The frequencies of the major (A, Ile) and minor (G, Val) alleles are shown in table 2.

### Demographic characteristics of the study population

The mean age at the onset of CML was found to be 37.5 years and ranged between 14 and 60 years. Male predominance has been observed in the present study with a sex ratio of 1.3:1 indicating that the male population are at higher risk for CML development. The results of our analysis of clinical characteristics in CML patients are presented in table 3.

**Table 1:** Distribution of CYP1A1*2C polymorphism genotypes in CML patients and controls


Genotype	Patients (n=132) n (%)	Controls (n=140) n (%)	OR	95% CI	P value

Ile/Ile (AA)- Wild	50 (38)	22 (16)	3.2705	1.84 - 5.81	0.0001
Ile/Val (AG)- Hetero	76 (57)	110 (78)	0.3701	0.218-0.63	0.0002
Val/Val (GG)- Homo mutant	6 (5)	8 (6)	0.7857	0.265-2.33	0.6635


CML; Chronic myeloid leukemia, n; Number of subjects, OR; Odds ratio and CI; Confidence interval.

**Table 2:** Allele frequencies of CYP1A1*2C polymorphism in CML patients and controls


Allele	Patients allele frequency	Controls allele frequency

Ile	0.67	0.55
Val	0.33	0.45


CML; Chronic myeloid leukemia.

**Table 3:** Association of CYP1A1*2C polymorphism in CML patients with demographic and clinical parameters


Characteristics	Total CML cases n=132 (%)	Wild type AA (Ile/Ile) n=51 (38%)	Heterozygous AG (Ile/Val) n=75 (58%)	Homo mutant GG (Val/Val) n=6 (4%)	P value

Gender					
Males	76 (58)	32 (42)	39 (51)	5 (6.6)	0.23
Females	56 (42)	19 (34)	36 (64)	1 (1.8)	0.23
Age of diagnosis (Y)					
<20	7 (5)	3 (43)	3 (43)	1 (14)	0.62
20-30	51 (39)	19 (37)	32 (63)	-	0.54
31-40	29 (22)	12 (41)	16 (55)	1 (3.4)	0.77
>40 (up to 60)	45 (34)	17 (38)	24 (53)	4 (8.8)	0.87
CML phase					
Chronic	114 (86)	46 (40)	62 (54)	6 (5)	0.24
Accelerated	13 (10)	3 (23)	10 (77)	-	0.18
Blast crisis	5 (4)	2 (40)	3 (60)	-	0.98
WBC count (cells/ cubic.mm of blood)					
<20 000	15 (11)	10 (67)	5 (33)	-	0.03*
>20 000	117 (89)	41 (35)	70 (60)	6 (5)	0.03*
Platelet count					
Normal count (1.5-4.0 lakhs cells/cubic.mm of blood)	71 (54)	28 (40)	38 (54)	5 (7)	0.64
Thrombocytopenia (<1.5 lakhs/cubic.mm)	3 (2.3)	2 (67)	1 (33)	-	0.37
Thrombocytosis (>4 lakhs/cubic.mm)	58 (44)	21 (36)	36 (62)	1 (1.7)	0.45
Spleen size					
Splenomegaly present	104 (79)	36 (35)	64 (62)	4 (3.8)	0.04*
Splenomegaly absent	28 (21)	15 (53.5)	11 (39)	2 (7)	0.04*


WBC; White blood corpuscles, CML; Chronic myeloid leukemia and *; Indicates statistically significant (P<0.05).

### Treatment response of chronic myeloid leukemia patients carrying the CYP1A*2C polymorphism

Association of the CYP1A1*2C polymorphism with respect to therapy responses, such as hematological and cytogenetic responses to imatinib treatment, are presented in table 4.

### Influence of the CYP1A1*2C polymorphism on chronic myeloid leukemia with respect to hematological response

MHR was observed to be higher in patients carrying the AG genotype (67%) than non-carriers (P=0.03), whereas, patients without the polymorphism (AA) (62%) exhibited a poorer hematological response than the patients with genotype AG (31%) (P=0.04), indicating that the patients carrying the polymorphism (AG) showed positive therapeutic response compared with wild type (AA) patients. Patients with the homozygous genotype (GG) (8%) exhibited poor hematological response (Table 4).

### Influence of CYP1A1*2C polymorphism on chronic myeloid leukemia with respect to cytogenetic response

We observed that most of the major cytogenetic responders (65%) carried a high frequency of Ile/ Val variant genotype, whereas most poor cytogenetic responders (78%) carried a high frequency of wild genotype (AA) (P=0.02). This indicates that this polymorphism may be associated with positive cytogenetic response to imatinib therapy. Patients with homozygous variant genotype (GG) (1%) exhibited major cytogenetic response (Table 4).

**Table 4:** Association of CYP1A1*2C polymorphism with therapeutic response in CML patients


Imatinib treatment response	Total n=95 n (%)	Total n=37 n (%) AA	Total n=57 n (%) AG	Total n=1 n (%) GG	P value

i. Hematological response					
Major (MHR)	70 (74)	23 (33)	47 (67)	-	0.03*
Minor (mHR)	12 (13)	6 (50)	6 (50)	-	0.42
Poor (PHR)	13 (14)	8 (62)	4 (31)	1 (8)	0.04*
ii.Cytogenetic response					
Major (MCyR)	71 (75)	24 (34)	46 (65)	1 (1)	0.08
Minor (mCyR)	15 (16)	6 (40)	9 (60)	-	0.90
Poor (pCyR)	9 (9)	7 (78)	2 (22)	-	0.02*


AA; Wild genotype, AG; Heterozygous genoype, GG; Homozygous variant genotype, MHR; Major hematological response, mHR; Minor hematological response, PHR; Poor hematological response, MCyR; Major cytogenetic response, mCyR; Minor cytogenetic response, pCyR; Poor cytogenetic response and *; Indicates statistically significant (P<0.05).

## Discussion

Polymorphisms in genes encoding the drug metabolizing enzyme *CYP1A1* contribute to the variability in susceptibility to various cancers (24, 26, 32). A few studies have reported a significant association of *CYP1A1* polymorphism with solid tumors (32), acute lymphoblastic leukemia (24) and CML (26). However, to our knowledge, not much information is available on the role of *CYP1A1* in relation to CML patients undergoing imatinib treatment. Generally, 95% of CML patients display genetic abnormality in the form of chromosomal translocation, i.e., Ph+ [t(9;22)], which causes the formation of the fusion oncogene **BCR-ABL**. The fusion protein formed from this fusion gene possesses enhanced tyrosine kinase activity which causes leukemogenesis (2).

Presence of the A4889G SNP in *CYP1A1* results in increased catalytic activity which leads to enhanced DNA adduct formation. These DNA adducts are responsible for causing mutations in the tumor suppressor genes and oncogenes, and thus trigger the uncontrolled hematopoietic cell proliferation, reduced differentiation and decreased apoptosis of malignant hematopoietic blast cells. Thus, individuals exhibiting an ability to activate procarcinogens due to sequence changes in *CYP1A1* may be at higher risk for various cancers including CML (26).

We found that although CML occurs at any age, a higher incidence was found in adults of age above 20 with most diagnosed to be in the chronic phase. CML patients are generally treated with the drug imatinib mesylate (Gleevec^®^, Novartis) with chronic phase CML patients responding well to this therapy. Hence it may be possible that SNPs in the *CYP1A1* may affect the response of the CML patients to imatinib therapy.

Our findings suggest that AG genotype may play a protective role in reducing CML risk in our cohort. We had also analysed the influence of CYP1A1*2C polymorphism with respect to various demographic and clinical characteristics of CML patients such as gender, age, CML phase, WBC count, platelet count and spleen size. With respect to gender we found that the males are more susceptible to developing CML than females. This may be attributed to the higher percentage of protective AG genotype in females and higher frequency of AA genotype in males of our study cohort. The frequency of AG genotype was higher than the AA wild genotype. This indicates that this SNP is not associated with the age of onset of CML.

We also found that the AG genotype was associated with good therapeutic response towards IM, which was measured in terms of hematological and cytogenetic responses, hence a good predictor of imatinib therapy.

Consistent with our findings, AML patients carrying the CYP1A1*2C polymorphism were reported to show a better prognosis compared with those carrying the wild-type genotype exhibiting a longer survival rate (33). Similarly, breast cancer patients carrying CYP1A1*2C polymorphism were shown to develop less aggressive tumors (34). Based on these reports, it is worth mentioning that the association of cancer with a particular polymorphism in one population may not be of the same significance in another population due to the variation in population demographics and other influencing factors such as the environment. Such results were also documented previously with respect to other genes including *MTHFR* (11).

Taspinar et al. (26) reported a higher distribution of *CYP1A1* Ile/Val heterozygous genotype in the Turkish CML patients (P<0.001) as compared with the controls, suggesting that carriers of *CYP1A1* Ile/Val (AG) genotype had an increased risk of developing CML. On the contrary, no association between the CYP1A1*2C polymorphism and CML risk was reported in the Iranian population (27). In addition, *CYP1A1* Val/Val homozygous variant genotype was associated with four-fold risk to ALL in Indian children (24). Some reports also suggested the association of CYP1A1*2C polymorphism with increased risk of solid tumors such as head and neck cancer in patients with Indian descent (17, 32). The above differences reported in the literature may be due to the differences in tumor type, ethnicity, environmental exposures, life style and habits.

Analysis of SNPs with respect to therapeutic response is expected to aid in designing novel, alternative therapeutic strategies to treat CML which may be efficient alone or may be useful in combination with existing therapies. With the advent of new techniques, obtaining the complete genomic sequence of every individual is becoming a possibility with which the SNPs in the genome could be identified and subsequently the risk of developing cancer may be predicted. In addition, the identified SNPs may not only serve as genetic markers in determining the susceptibility of an individual to a disease, it can also predict response to a therapy. Such advances are expected to pave the way for the development of personalized medicine.

## Conclusion

We showed that the AG genotype of CYP1A1*2C polymorphism may play a protective role against CML. Our analysis on influence of this polymorphism on therapy of CML patients of Indian descent suggests that this genotype serves as a good predictor to assess response to imatinib therapy.
